# Estimation of the Impact of Abdominal Adipose Tissue (Subcutaneous and Visceral) on the Occurrence of Carbohydrate and Lipid Metabolism Disorders in Patients with Obesity—A Pilot Study

**DOI:** 10.3390/nu16091301

**Published:** 2024-04-26

**Authors:** Katarzyna Witczak-Sawczuk, Lucyna Ostrowska, Urszula Cwalina, Joanna Leszczyńska, Marta Jastrzębska-Mierzyńska, Marcin Krzysztof Hładuński

**Affiliations:** 1 Department of Dietetics and Clinical Nutrition, Medical University of Bialystok, ul. Mieszka I 4 B, 15-054 Bialystok, Poland; katarzyna.witczak-sawczuk@umb.edu.pl (K.W.-S.);; 2 Department of Biostatistics and Medical Informatics, Medical University of Bialystok, ul. Szpitalna 37, 15-295 Bialystok, Poland; 3 Independent Laboratory of Molecular Imaging, Medical University of Bialystok, ul. Zurawia 71A, 15-540 Bialystok, Poland

**Keywords:** adipose tissue, obesity, metabolic risk

## Abstract

Obesity represents a significant global public health concern. The excessive accumulation of abdominal adipose tissue is often implicated in the development of metabolic complications associated with obesity. Our study aimed to investigate the impact of particular deposits of abdominal adipose tissue on the occurrence of carbohydrate and lipid metabolism complications. We established cut-off points for visceral adipose tissue (VAT), subcutaneous adipose tissue (SAT), and the VAT/SAT ratio at which selected metabolic complications of obesity-related diseases (disorders of carbohydrate and/or lipid metabolism) occur. We conducted an observational study involving 91 subjects with first- and second-degree obesity, accounting for gender differences. Anthropometric measurements were taken, body composition analysis (BIA) was conducted, and biochemical determinations were made. Our findings suggest that commonly used parameters for assessing early metabolic risk, such as BMI or waist circumference, may overlook the significant factor of body fat distribution, as well as gender differences. Both visceral and subcutaneous adipose tissue were found to be important in estimating metabolic risk. We identified the cut-off points in women in terms of their elevated fasting glucose levels and the presence of insulin resistance (HOMA-IR: homeostasis model assessment of insulin resistance) based on SAT, VAT, and the VAT/SAT ratio. In men, cut-off points were determined for the presence of insulin resistance (HOMA-IR) based on VAT and the VAT/SAT ratio. However, the results regarding lipid disorders were inconclusive, necessitating further investigation of a larger population.

## 1. Introduction

Obesity currently stands as a prominent worldwide public health issue. The World Health Organization defines it as a complex condition associated with the abnormal buildup of excess fat in the human body, significantly elevating the likelihood of various non-communicable diseases [1]. This condition is chronic and progressive, lacking spontaneous resolution, and is characterized by a tendency to recur [2].

Visceral fat tissue is often implicated as a key factor in the onset of metabolic complications associated with obesity [3]. This is attributed to the higher concentrations of angiotensinogen (a precursor to angiotensin) and leptin (a hormone regulating appetite) found in subcutaneous fat tissue [4,5]. In contrast, visceral fat tissue is characterized by elevated lipolytic activity, resulting in the release of significant amounts of free fatty acids (FFAs), increased gluconeogenesis, and heightened secretion of pro-inflammatory adipokines such as interleukin 6 (IL-6), resistin, visfatin, and TNF-α [5]. The current scientific findings indicate that visceral distribution of adipose tissue is associated with an increased risk of metabolic disorders, including abnormalities in carbohydrate metabolism such as insulin resistance and/or hyperglycemia. These conditions may lead to the development of type 2 diabetes, hypertension, or ischemic heart disease [6]. Consequently, visceral adipose tissue (VAT) is now acknowledged as more metabolically active and poses a greater metabolic and inflammatory risk compared to subcutaneous adipose tissue (SAT) [7,8,9]. However, it is essential to note that the distribution of fat tissue can vary among individuals. Additionally, subcutaneous fat tissue may exist as a superficial layer (SSAT) or a deep layer (DSAT), which shares metabolic similarities with visceral fat tissue [10].

In 2009, researchers, utilizing body scanning diagnostic techniques, illustrated the lack of consistency and predictability in the distribution of fat tissue. They observed that in a portion of the population, adipose tissue is predominantly located in the visceral area, with only limited subcutaneous presence. Conversely, another group of individuals exhibited a substantial deposit of subcutaneous fat and a minimal amount of visceral fat [11]. The reasons behind such a varied distribution of adipose tissue in the population, whether modifiable or non-modifiable, have yet to be fully understood. The author’s own research has delved into an investigation of whether and to what extent individual deposits of adipose tissue influence the occurrence of disorders in carbohydrate and lipid metabolism, both in women and men.

The author’s research examined the impact of individual deposits of abdominal fat tissue, including visceral and subcutaneous fat, as well as the VAT/SAT ratio, on the development of specific complications in carbohydrate and lipid metabolism. The study aimed to determine the threshold levels of visceral and subcutaneous fat tissue and the VAT/SAT ratio at which the onset of selected obesity-related complications is observed.

## 2. Materials and Methods

This study was the result of a research project that was registered at www.clinicaltrials.gov under the number NCT05612282; (accessed on 11 April 2022).

### 2.1. Ethics

The research procedure was approved by the Bioethics Committee of the Medical University of Białystok, approval numbers R-I-002/442/2015 and APK.002.528.2022. Each patient qualified for the study received detailed information about the course of the study and signed informed consent to participate in the study.

### 2.2. Study Principants

The observational study comprised a total of 91 individuals with obesity class I and II, based on their BMI. Among them, 64.8% were women (*n* = 59), and 35.2% were men (*n* = 32). All the participants met the complete inclusion criteria for the study, and no exclusion criteria were identified. The research project enrolled individuals of both genders, aged 20–65, with primary obesity (BMI ≥ 30 kg/m^2^–≤39.99 kg/m^2^). The exclusion criteria included secondary obesity, a BMI ≥ 40 kg/m^2^, previous surgical interventions for obesity, and having undergone other surgical procedures (at least 3 months prior). Additionally, the exclusion criteria included those with type 2 diabetes or insulin resistance (previously diagnosed or identified during therapy), hormonal disorders, hormonal contraception or hormone replacement therapy, prior oral steroid therapy, antiretroviral therapy, eating disorders, aggravated coronary heart disease, patients with pacemakers, and pregnant or lactating women.

### 2.3. Antropometric Parameters

The assessment of body weight involved the use of an electronic RADWAG medical scale equipped with a height meter (WPT 60/150 OW). The patients’ heights were measured with a 0.1 cm margin of error, and body weight was measured with a 0.1 kg margin of error. To ensure accurate measurements, the patients were requested to remove their shoes and outer clothing.

Subsequently, the body mass index (BMI) was individually calculated for each patient by dividing their body weight, expressed in kilograms, by their height, expressed in meters squared. In accordance with the inclusion criteria, patients with a BMI ranging from ≥30 kg/m^2^ to ≤39.9 kg/m^2^ were deemed eligible for the study [12,13].

The waist circumference measurements were conducted using a metric tape with an accuracy of 0.5 cm. The waist circumference measurements were taken midway between the lower costal arch and the upper edge of the ilium in the mid-axillary line, and the results were expressed in centimeters [3,13].

### 2.4. Body Analysis

The body composition analysis utilized the bioelectrical impedance method (BIA) using a BioScan 920-2 device (Essex, UK). Each patient received information regarding the necessary criteria for study inclusion—the analysis was conducted on an empty stomach with the bladder emptied 30 min prior to the examination; a 12 h refrain from physical activity was required prior to the examination; the elimination of alcohol and caffeinated beverages from the diet 24 h before the examination was recommended; individuals with a pacemaker were excluded from participation [14].

To determine the areas of subcutaneous and visceral fat tissue, the BIA method was employed using the BioScan 920-2 device (Essex, UK), and the following measurements were estimated [14]:

- Visceral fat tissue area (cm^2^) at the height of the measurement (at the navel);
- Subcutaneous fat tissue area (cm^2^) at the height of the measurement (at the navel);
- VAT/SAT ratio—the ratio of visceral fat tissue to subcutaneous fat tissue.

The obtained results were processed using the Maltron BioScan 920 v1.1 software.

### 2.5. Biochemical Analysis

The evaluation of specific biochemical parameters for each participant was performed once at the Medical Laboratory within the University Clinical Hospital in Białystok in accordance with the established standards. Venous blood (15 mL) was collected from the antecubital vein. Following the collection, the blood underwent centrifugation to obtain serum. The concentrations of the following parameters were assessed in the blood serum: fasting glucose, fasting insulin, total cholesterol, LDL fraction cholesterol, HDL fraction cholesterol, and triglycerides. The reference values for fasting insulin concentration were set at <10 mU/mL. The fasting glucose and fasting insulin results were used to calculate the homeostasis model assessment of insulin resistance (HOMA-IR) (fasting insulin concentration (mU/mL) × fasting glucose concentration (mmol/L)/22.5). HOMA-IR values ≥ 2 indicated tissue resistance to insulin. This parameter was used to assess the occurrence of insulin resistance and, consequently, to assess the risk of developing metabolic syndrome [15]. To determine the non-HDL cholesterol fraction, the following mathematical operation was performed: total cholesterol concentration—HDL fraction cholesterol [16].

### 2.6. Statistical Analysis

The statistical analysis of the collected data was conducted using the STATISTICA 13.3 program (StatSoft, Kraków, Poland). The participants were categorized into groups based on their gender. The study variables were described using medians and the first and third quartiles. Given the non-normal distribution of the data, non-parametric statistical methods were employed, with the normality assessed using the Shapiro–Wilk test. Spearman’s correlation coefficient was utilized to assess the relationships between quantitative variables. Cut-off points were determined for the visceral fat tissue area (evaluated at the level of the navel), subcutaneous fat tissue area (also assessed at the level of the navel), and the VAT/SAT ratio concerning all the examined biochemical parameters in the participants. The relationship between the continuous and binary variables was explored using ROC curves. The selection of the most accurate predictors involved assessing the AUC (Area Under the Curve) values. The optimal cut-off points were identified using the tangent method and Youden’s index. For all the results obtained in this study, *p* < 0.05 was considered statistically significant.

## 3. Results

### 3.1. Characteristics of the Study Group of Men and Women in Terms of Anthropometric Parameters

The research comprised 59 female and 32 male participants. The median age of the study group was 47 years for women and 42 years for men. Approximately 75% of the female participants had class I obesity, whereas among the male participants, 50% had class I obesity, and the other 50% had class II obesity. The characteristics of both the female and male participants concerning the selected anthropometric parameters and their body composition are detailed in Table 1.

Analyzing the impact of the studied factors on the carbohydrate metabolism parameters (Table 2) revealed that in women, an increase in fasting glucose concentration correlated with higher values for age (r = 0.41, *p* = 0.001), body weight (r = 0.25, *p* = 0.04), waist circumference (r = 0.28, *p* = 0.02), total body fat (r = 0.28, *p* = 0.03), percentage of adipose tissue (r = 0.32, *p* = 0.01), visceral adipose area at the umbilicus (r = 0.26, *p* = 0.04), and subcutaneous adipose area at the umbilicus (r = 0.26, *p* = 0.04). Conversely, in men, only age significantly influenced fasting glucose (r = 0.41, *p* = 0.01). No significant effect of the studied parameters on the fasting insulin concentrations was observed in women, whereas in men, their fasting insulin concentration increased with a higher body weight (r = 0.35, *p* = 0.04), BMI (r = 0.37, *p* = 0.03), waist circumference (r = 0.35, *p* = 0.04), and total body fat (r = 0.36, *p* = 0.04). Furthermore, in women, their HOMA-IR values were significantly influenced by percentage of adipose tissue (r = 0.29, *p* = 0.02) and subcutaneous adipose area (r = 0.27, *p* = 0.03), whereas no such correlations were observed in men.

Additionally, it was noted (Table 3) that in women, the VAT area (r = −0.32, *p* = 0.01) and the VAT/SAT ratio (r = −0.32, *p* = 0.01) were associated with reduced HDL cholesterol concentrations, while an increase in triglycerides was linked to an increase in the VAT area (r = 0.26, *p* = 0.04). However, no such correlations were evident in men.

### 3.2. Estimation of Cut-Off Points for the Area of Visceral and/or Subcutaneous Adipose Tissue at Which Selected Parameters of Carbohydrate and/or Lipid Metabolism Occurred in Obese Participants

**Figure 1:**
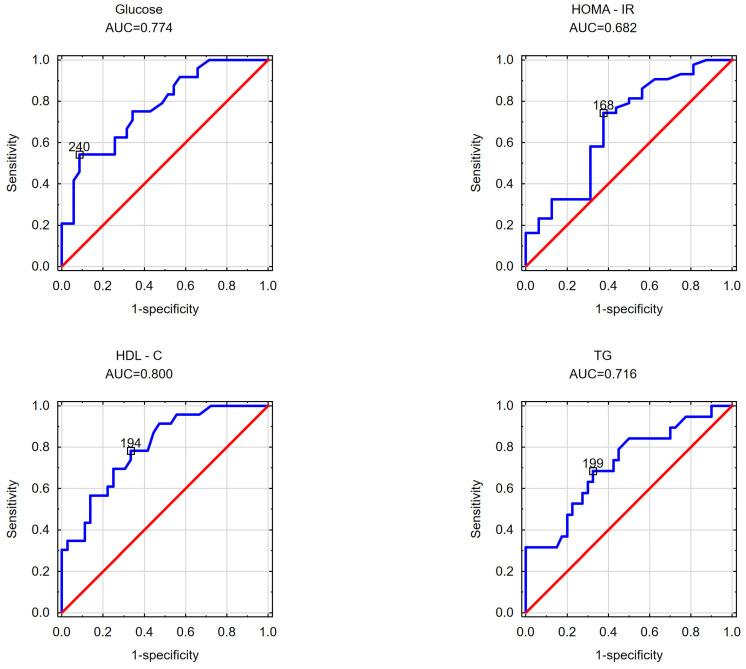
Cut-off points of the visceral fat tissue area estimated at the navel at which increased fasting glucose levels, increased HOMA-IR values, decreased HDL cholesterol levels, and increased triglyceride levels were observed in the study group of women.

**Figure 2:**
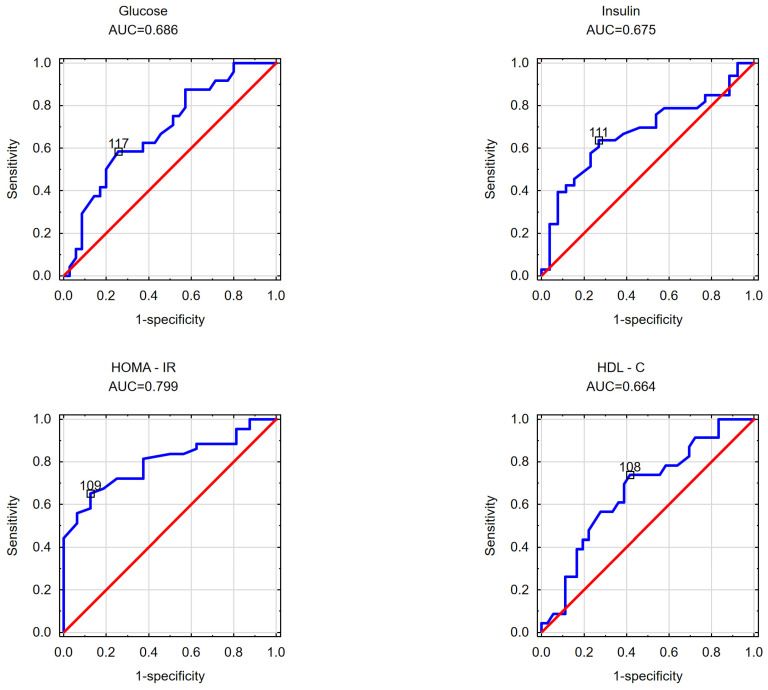
Illustrates the threshold values of the subcutaneous adipose area estimated at the navel, where elevated fasting glucose levels, heightened fasting insulin levels, increased HOMA-IR values, and reduced HDL cholesterol levels were observed in the female cohort.

**Figure 3:**
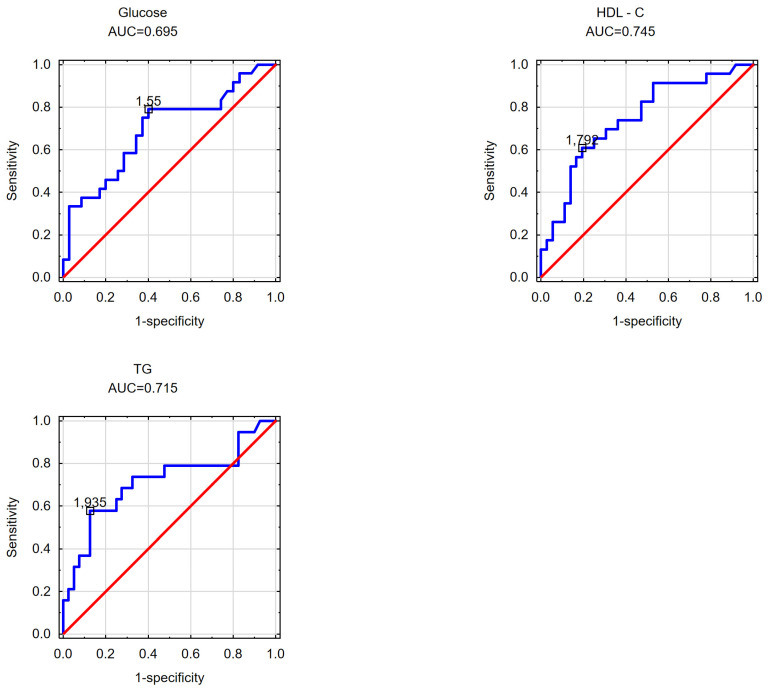
VAT/SAT ratio cut-off points at which increased fasting glucose levels, decreased HDL cholesterol levels and increased triglyceride levels in the study group of women were detected.

**Figure 4:**
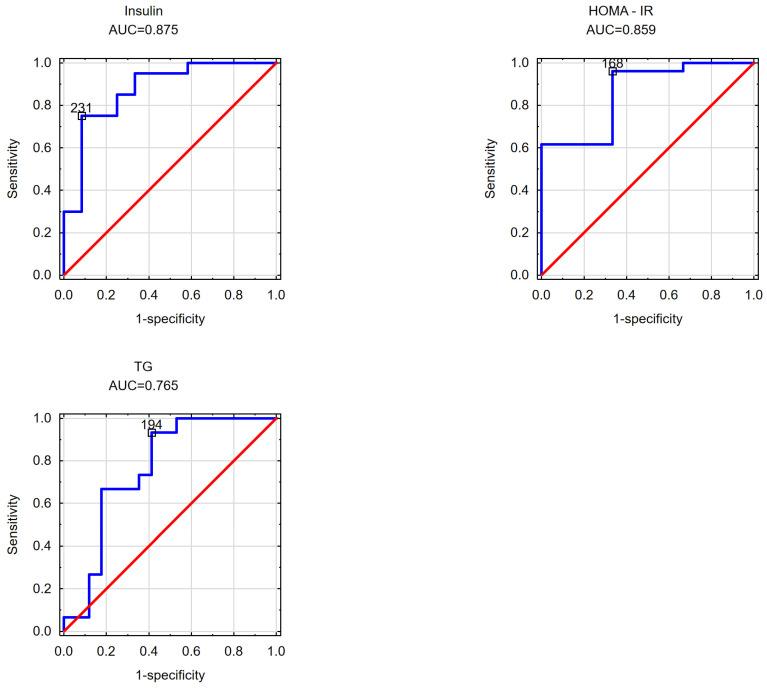
Cut-off points of the visceral adipose tissue area estimated at the navel at which increased fasting insulin levels, an elevated HOMA-IR index, and elevated triglyceride levels were observed in the study group of men.

**Figure 5:**
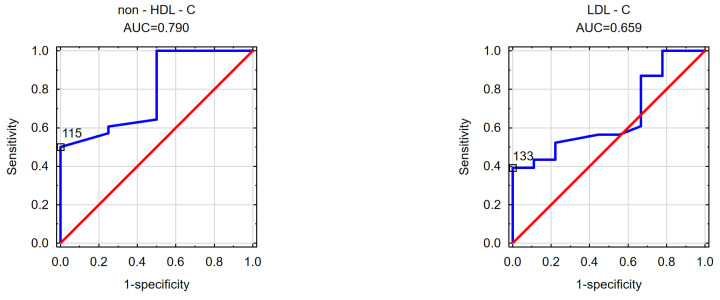
Cut-off points of the subcutaneous adipose tissue area estimated at the navel at which an increased non-HDL cholesterol fraction and an increased LDL cholesterol fraction were observed in the male cohort.

**Figure 6:**
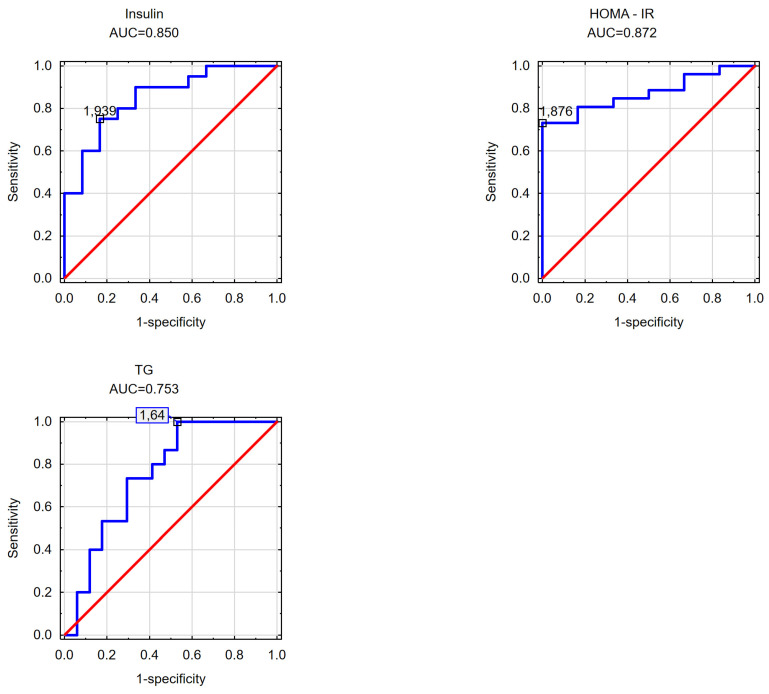
VAT/SAT ratio cut-off points at which increased fasting insulin concentration, elevated HOMA-IR index values, and elevated triglyceride concentrations were observed in the male cohort.

In the subsequent phase of the study, an effort was made to determine the cut-off values for the visceral adipose area measured at the umbilicus level beyond which disorders in selected parameters of carbohydrate and/or lipid metabolism occurred in the obese patients. Likewise, the cut-off values for the subcutaneous adipose tissue area measured at the umbilicus level and the VAT/SAT ratio were analyzed. The findings are detailed in Table 4 and Table 5 and Figure 1, Figure 2, Figure 3, Figure 4, Figure 5 and Figure 6.

Upon evaluating the cut-off values for visceral adipose tissue and the examined parameters among the female cohort, it was determined that the threshold for insulin resistance (HOMA-IR) was 168 cm^2^, while for abnormally elevated fasting blood glucose, it was 240 cm^2^. The cut-off value for visceral adipose tissue and decreased HDL cholesterol concentration was identified as 1194 cm^2^, whereas for elevated serum triglycerides, it was 199 cm^2^. These findings are illustrated in Figure 1.

In women, it was determined that the cut-off for the subcutaneous adipose area (as depicted in Figure 2) measured at the umbilicus was 117 cm^2^ for elevated fasting glucose concentration, 111 cm^2^ for a fasting insulin concentration ≥ 10 mU/mL, 109 cm^2^ for the presence of insulin resistance (HOMA-IR), and 108 cm^2^ for a reduced HDL cholesterol fraction concentration.

Figure 3 illustrates the estimated threshold values for the VAT/SAT ratio in women with elevated fasting glucose levels (1.55), reduced HDL cholesterol fraction levels (1.79), and elevated serum triglycerides (1.95).

Determining the cut-off values for visceral adipose tissue and the measured parameters in the male cohort revealed that it was 231 cm^2^ for a fasting insulin concentration of ≥10 mU/mL, 168 cm^2^ for the presence of insulin resistance, and 194 cm^2^ for the presence of hypertriglyceridemia. These findings are depicted in Figure 4.

Figure 5 displays the estimated cut-off points for the subcutaneous adipose tissue (SAT) area in men for elevated non-HDL cholesterol (115 cm^2^) and for elevated LDL cholesterol fractions (133 cm^2^).

In the male group, cut-off points for the VAT/SAT ratio were determined for the presence of hypertriglyceridemia (1.64), for the presence of insulin resistance (1.876), and for a fasting insulin concentration ≥ 10 mU/mL (1.939). These results are illustrated in Figure 6.

## 4. Discussion

Obesity presents as a significant independent risk factor for metabolic complications [17]. Central to obesity is the excessive buildup of adipose tissue due to a positive energy balance [18,19]. It is essential to recognize that adipose tissue functions as an endocrine organ, prone to significant proliferation, potentially comprising a substantial portion of human body weight, even up to 70% [20]. The surplus accumulation of adipose tissue increases the risk of chronic ailments, including cardiovascular diseases, disturbances in carbohydrate metabolism, cancer (especially gastrointestinal-related types), and joint disorders (largely attributed to mechanical stress on the joints) [18].

Until recently, obesity was categorized into gynoid and android types based on fat distribution. Gynoid obesity primarily involves fat accumulation in the gluteal–femoral region [19,21]. Conversely, android (central) obesity is characterized by abdominal fat accumulation, which is often implicated in the onset of metabolic disorders [21]. Adipose tissue located centrally is further classified into subcutaneous and visceral compartments. Visceral adipose tissue in particular has been consistently identified as a contributor to the development of metabolic complications associated with obesity [19]. Given this context, it is logical to explore the question of the factors contributing to the development of obesity-related complications, with a specific focus on the role of different adipose tissue distributions. Hence, it becomes essential to analyze abdominal adipose tissue deposits separately, distinguishing between visceral and subcutaneous fat.

The body mass index (BMI) is the most commonly utilized parameter for diagnosing obesity and in obesity-related research. However, despite its consideration of body weight and height, BMI has limitations as a predictor of metabolic disorder risk. These limitations stem from its failure to account for adipose tissue content and distribution, which are recognized as significant predictors of metabolic complications associated with obesity. While an updated version of the obesity diagnosis criteria based on BMI was introduced by the American Endocrine Societies in 2016, it primarily focused on specific complications rather than their risk factors [22]. Moreover, Stoklossa et al. observed that BMI overestimated the lean body mass in patients with a BMI > 35 kg/m^2^ when using body composition assessment according to bioelectrical impedance analysis (BIA). In contrast, assessments using dual-energy X-ray absorptiometry (DXA) yielded more accurate and reliable results [23].

Waist circumference serves as a crucial parameter acknowledged by the WHO for assessing abdominal obesity and the heightened risk of developing metabolic disorders in individuals with obesity [3]. However, it is noteworthy that the standards for this parameter are rather diverse. According to the IDF, the presence of central obesity is indicated by waist circumferences ≥ 80 cm in women and ≥94 cm in men. Nevertheless, these values do not provide insight into the obesity phenotype [24]. It might be more appropriate to evaluate waist circumference based on the NCEP-ATP III criteria, which incorporate higher thresholds for women (≥88 cm) and men (≥102 cm) [25]. However, even with an adjustment in the reference values for waist circumference, differences in the distribution of visceral and subcutaneous fat within the abdominal cavity remain unaddressed. Therefore, while waist circumference serves as a predictive parameter for metabolic disorder development in obesity, it is prudent to seek more sensitive parameters for estimating metabolic risk in obese individuals.

Our research aimed to identify methods for assessing early metabolic risk in individuals with obesity. We sought to establish the cut-off points for visceral adipose tissue, subcutaneous adipose tissue at the umbilical level, and the VAT/SAT ratio in relation to parameters of carbohydrate and lipid metabolism. The factors contributing to the preferential accumulation of visceral adipose tissue or subcutaneous adipose tissue remain incompletely understood. Therefore, when discussing abdominal obesity and adipose tissue accumulation in the abdominal area, it is prudent to examine which adipose tissue deposits may cause predisposition to the earlier development of metabolic complications associated with obesity. This analysis includes consideration of whether visceral adipose tissue alone, subcutaneous adipose tissue alone (with particular focus on deep subcutaneous adipose tissue), or the ratio between the visceral adipose tissue and the subcutaneous adipose tissue (VAT/SAT ratio) is significant [26,27].

When analyzing the impact of adipose tissue distribution on metabolic disorders, gender is a critical factor to consider. This is particularly pertinent when evaluating parameters such as waist circumference or total body fat percentage, as well as in biochemical analyses like HDL fraction cholesterol. Gender significantly influences the assessment of the risk of specific metabolic complications and guides the selection of therapeutic approaches to the complications arising from obesity [28].

The prevailing studies in the available literature have primarily focused on establishing cut-off points for BMI or waist circumference concerning the risk of metabolic parameter abnormalities [29,30]. Additionally, certain researchers have sought cut-off points for visceral adipose tissue, indicating significant increases in the risk of metabolic complications linked to obesity [31,32]. A notable challenge in comparing these studies with our research lies in the variations in the visceral fat assessment methods (DXA, BIA, CT), leading to different units for estimating its content (cm^2^, cm^3^, grams). For instance, one study observed that the overall metabolic risk in postmenopausal women escalated with VAT surpassing 117.8 cm^2^ [31]. Notably, an insufficient number of analyses determining cut-off points for VAT, SAT, and the VAT/SAT ratio concerning individual obesity-related disorders exists.

According to our study findings, subcutaneous adipose tissue emerges as a significant factor in assessing the risk of developing carbohydrate-related complications in women, with lower cut-off points compared to those for visceral adipose tissue. Specifically, in women, the cut-off point for elevated fasting glucose levels was 117 cm^2^ for subcutaneous adipose tissue, whereas for visceral adipose tissue, it was 240 cm^2^. Similarly, insulin resistance (HOMA-IR) and abnormal fasting glucose were detected at lower subcutaneous adipose tissue thresholds (109 cm^2^) compared to visceral adipose tissue (168 cm^2^).

Conversely, in men, visceral fat and its area seem to be associated with the onset of carbohydrate-related complications, and cut-off points for subcutaneous adipose tissue were not established. Notably, the cut-off point for visceral adipose tissue indicating the presence of insulin resistance (HOMA-IR ≥ 2) was identical between men and women, at 168 cm^2^. The identification of the visceral adipose tissue cut-off point for insulin resistance underscores the significance of this metric in early risk assessment for obesity-related complications in men.

Conversely, for lipid disorders in women, the findings obtained require further clarification through a study involving a larger cohort. In determining the cut-off points for a decreased HDL cholesterol fraction, it was noted that the cut-off point for subcutaneous adipose tissue (108 cm^2^) was lower than that for visceral adipose tissue (194 cm^2^). However, it was observed that the cut-off point for visceral adipose tissue had a higher AUC (AUC = 0.800) compared to the cut-off point for subcutaneous adipose tissue (AUC = 0.664). Nevertheless, these findings do not provide conclusive evidence. For elevated triglycerides, the cut-off point for visceral adipose tissue was determined to be 199 cm^2^, while no statistically significant cut-off point for elevated TGs was identified for subcutaneous adipose tissue. Similarly, in men, the results regarding lipid disorders are inconclusive. It appears that each parameter needs to be individually analyzed. Abnormal triglyceride concentrations in men seem to be associated with visceral adipose tissue accumulation (168.5 cm^2^). Conversely, elevated concentrations of non-HDL cholesterol (115 cm^2^) and LDL cholesterol (133 cm^2^) in men may be linked to subcutaneous adipose tissue distribution. However, these analyses warrant validation in a larger cohort. The study results underscore the importance of considering gender disparities in future investigations, as men and women exhibit variations in the distribution of both subcutaneous and visceral adipose tissue. This is evident from the disparate VAT and SAT cut-off points observed in the analyzed parameters of carbohydrate and lipid disorders.

In our study design, we aimed to develop practical methods for the early detection of metabolic risk applicable in routine patient care. While dual-energy X-ray absorptiometry (DXA) is considered the gold standard for assessing total adipose tissue volume (both subcutaneous and visceral), we opted for bioelectrical impedance analysis (BIA) to evaluate total visceral adipose tissue (VAT) and subcutaneous adipose tissue (SAT) due to its practical feasibility. An important aspect of our research is the gender stratification of the study population given the variations in the total adipose tissue percentage norms and the reference values for the HDL cholesterol concentrations between men and women. However, the small sample sizes, particularly in the male subgroup (*n* = 32), represent a limitation of this pilot study. Nonetheless, it should be emphasized that this study serves as an initial exploration in this field.

## 5. Conclusions

The global prevalence of obesity underscores the need for novel, precise parameters to assess early metabolic risk in patients. Current metrics like body mass index (BMI) or waist circumference lack consideration of the adipose tissue distribution and gender disparities. Our study explored the utility of bioelectrical impedance analysis (BIA) of the subcutaneous adipose tissue at the waist height, visceral adipose tissue (VAT), and the VAT/SAT ratio in predicting carbohydrate and lipid disorders. In women, the cut-off points for elevated fasting glucose (≥100 mg/dL) were determined as 117 cm^2^ for SAT, 240 cm^2^ for VAT, and 1.55 for the VAT/SAT ratio. For a HOMA-IR score ≥ 2, the cut-off points were 109 cm^2^ for SAT and 168 cm^2^ for VAT. Conversely, in men, only the VAT cut-off point for a HOMA-IR score ≥2 was established at 168 cm^2^ and that for the VAT/SAT ratio at 1.876.

Regarding lipid disorders, the cut-off point for elevated triglyceride levels (>150 mg/dL) was determined to be 199 cm^2^ for VAT in women and 168.5 cm^2^ in men. Furthermore, the cut-off point for an elevated TG concentration relative to the VAT/SAT ratio was found to be 1.935 in women and 1.64 in men. For reduced HDL-C (<50 mg/dL), the cut-off points were established at 194 cm^2^ for VAT, 108 cm^2^ for SAT, and 1.79 for the VAT/SAT ratio in women. However, these findings warrant validation through larger-scale studies involving individuals with obesity.

## Figures and Tables

**Table 1:** Participant characteristics.

	*n*	Age Me (Q1–Q3)	Body Weight Me (Q1–Q3)	BMI Me (Q1–Q3)	WC Me (Q1–Q3)	BF (kg) Me (Q1–Q3)	PBF (%) Me (Q1–Q3)	VAT (cm^2^) Me (Q1–Q3)	SAT (cm^2^) Me (Q1–Q3)	VAT/SAT Me (Q1–Q3)
Women	59	47.0 (39.0–57.0)	90.0 (84.5–96.7)	33.6 (32.0–35.1)	109.0 (104.0–115.0)	39.9 (35.7–43.6)	43.9 (42.2–46.2)	198.0 (148.0–262.0)	112.0 (101.0–136.0)	1.70 (1.28–2.08)
Men	32	42.0 (36.5–48.5)	115.0 (103.5–124.0)	35.2 (32.3–37.3)	120.0 (113.2–123.5)	40.2 (33.5–44.2)	34.4 (32.5–37.0)	231.5 (183.5–324.0)	113.0 (101.5–134.5)	2.09 (1.70–2.43)

BMI—body mass index; Me—median; Q1—quartile 1; Q3—quartile 3; WC—waist circumference; BF—body fat; PBF—percentage of body fat; VAT—visceral adipose tissue; SAT—subcutaneous adipose tissue; VAT/SAT—visceral-to-subcutaneous-fat ratio.

**Table 2:** Assessment of the correlation between age and specific anthropometric and body composition parameters among patients and carbohydrate metabolism parameters.

		Age	Body Weight	BMI	WC	BF (kg)	PBF (%)	VAT (cm^2^)	SAT (cm^2^)	VAT/SAT
WOMEN										
Fasting glucose	r	0.41 *	0.25 *	0.24	0.28 *	0.28 *	0.32 *	0.26 *	0.26 *	0.16
	*p*	0.001	0.04	0.06	0.02	0.03	0.01	0.04	0.04	0.19
Fasting insulin	r	0.16	0.04	0.08	0.10	0.07	0.24	0.14	0.24	0.08
	*p*	0.21	0.74	0.52	0.40	0.56	0.06	0.27	0.06	0.52
HOMA-IR	r	0.23	0.08	0.11	0.14	0.12	0.29 *	0.18	0.27 *	0.11
	*p*	0.07	0.55	0.37	0.28	0.35	0.02	0.16	0.03	0.38
MEN										
Fasting glucose	r	0.41 *	−0.26	−0.12	−0.14	−0.15	0.00	0.10	−0.10	0.07
	*p*	0.01	0.13	0.48	0.41	0.40	0.98	0.95	0.56	0.69
Fasting insulin	r	0.01	0.35 *	0.37 *	0.35 *	0.36 *	0.23	0.25	0.23	0.14
	*p*	0.93	0.04	0.03	0.04	0.04	0.20	0.16	0.18	0.43
HOMA-IR	r	0.13	0.18	0.23	0.23	0.22	0.19	0.26	0.21	0.15
	*p*	0.45	0.31	0.19	0.18	0.22	0.29	0.14	0.22	0.38

* *p* < 0.05,—statistically significant; BMI—body mass index; WC—waist circumference; BF—body fat; PBF—percentage of body fat; VAT—visceral adipose tissue; SAT—subcutaneous adipose tissue; VAT/SAT—visceral-to-subcutaneous-fat ratio; HOMA-IR—homeostasis model assessment of insulin resistance.

**Table 3:** Evaluation of the association between age and specific anthropometric parameters and body composition among patients and lipid metabolism parameters.

		Age	Body Weight	BMI	WC	BF (kg)	PBF (%)	VAT (cm^2^)	SAT (cm^2^)	VAT/SAT
WOMEN										
Total cholesterol	r	0.19	−0.07	−0.01	0.05	0.02	0.12	0.08	0.05	0.05
	*p*	0.13	0.57	0.88	0.68	0.85	0.33	0.52	0.70	0.70
LDL-C	r	0.05	0.00	0.02	0.02	0.05	0.07	0.11	0.08	0.04
	*p*	0.69	0.95	0.83	0.84	0.69	0.57	0.39	0.54	0.73
HDL-C	r	−0.09	−0.12	−0.07	−0.06	−0.17	−0.20	−0.32 *	−0.12	−0.32 *
	*p*	0.46	0.34	0.58	0.60	0.19	0.11	0.01	0.33	0.01
Non-HDL-C	r	0.19	−0.05	0.01	0.05	0.05	0.17	0.14	0.07	0.12
	*p*	0.13	0.69	0.92	0.68	0.65	0.17	0.26	0.56	0.34
Triglycerydes (TGs)	r	0.16	0.06	0.11	0.12	0.13	0.18	0.26 *	0.09	0.23
	*p*	0.20	0.61	0.38	0.32	0.32	0.15	0.04	0.48	0.07
MEN										
Total cholesterol	r	0.32	−0.15	−0.08	−0.04	0.01	0.12	−0.00	0.14	−0.03
	*p*	0.07	0.41	0.66	0.72	0.92	0.52	0.99	0.43	0.83
LDL-C	r	0.20	0.01	−0.05	0.03	0.09	0.09	0.00	0.13	−0.11
	*p*	0.27	0.92	0.77	0.82	0.61	0.62	0.99	0.45	0.51
HDL-C	r	−0.00	−0.21	−0.04	−0.14	−0.09	−0.05	−0.25	−0.19	−0.10
	*p*	0.99	0.23	0.81	0.43	0.62	0.75	0.15	0.28	0.55
Non-HDL-C	r	0.32	−0.07	−0.02	0.02	0.08	0.17	0.05	0.20	−0.03
	*p*	0.06	0.70	0.87	0.90	0.65	0.36	0.77	0.28	0.84
Triglycerydes (TGs)	r	0.27	−0.01	0.01	0.03	0.01	0.07	0.22	0.14	0.29
	*p*	0.12	0.93	0.95	0.85	0.94	0.69	0.21	0.43	0.10

* *p* < 0.05,—statistically significant; BMI—body mass index; WC—waist circumference; BF—body fat; PBF—percentage of body fat; VAT—visceral adipose tissue; SAT—subcutaneous adipose tissue; VAT/SAT—visceral-to-subcutaneous-fat ratio; LDL—low-density lipoprotein; HDL—high-density lipoprotein.

**Table 4:** VAT, SAT, and VAT/SAT ratio cut-off points at which increased values of selected carbohydrate metabolism parameters in the studied groups of women and men were detected.

WOMEN		
VAT	SAT	V/S ratio
glucose AUC = 0.774 cut-off point = 240 cm^2^	glucose AUC = 0.686 cut-off point = 117 cm^2^	glucose AUC = 0.695 cut-off point = 1.55
HOMA-IR AUC = 0.682 cut-off point = 168 cm^2^	insulin AUC = 0.675 cut-off point = 111 cm^2^	
	HOMA-IR AUC = 0.799 cut-off point = 109 cm^2^	
MEN		
VAT	SAT	V/S ratio
insulin AUC = 0.875 cut-off point = 231 cm^2^		insulin AUC = 0.850 cut-off point = 1.939
HOMA-IR AUC = 0.859 cut-off point = 168 cm^2^		HOMA-IR AUC = 0.872 cut-off point = 1.876

**Table 5:** VAT, SAT, and VAT/SAT ratio cut-off points at which increased values of selected lipid metabolism parameters in the studied groups of women and men were detected.

WOMEN		
VAT	SAT	V/S Ratio
HDL-C AUC = 0.800 cut-off point = 194 cm^2^	HDL-C AUC = 0.664 cut-off point = 108 cm^2^	HDL-C AUC = 0.745 cut-off point = 1.79
TGs AUC = 0.716 cut-off point = 199 cm^2^		TGs AUC = 0.715 cut-off point = 1.935
MEN		
VAT	SAT	V/S ratio
TGs AUC = 0.765 cut-off point = 168.50 cm^2^	non-HDL-C AUC = 0.790 cut-off point = 115 cm^2^	TGs AUC = 0.753 cut-off point = 1.64
	LDL—C AUC = 0.659 cut-off point = 133 cm^2^	

## Data Availability

Data are available on request due to privacy/ethics restrictions.
